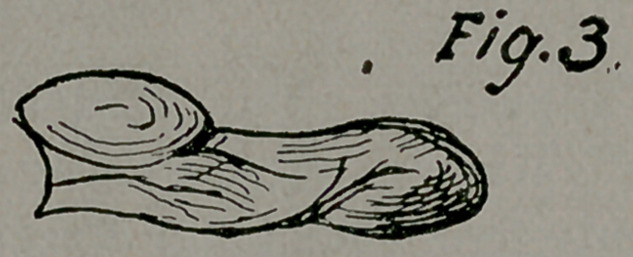# Some Unusual Cases in Recent Abdominal Work

**Published:** 1892-07

**Authors:** T. J. Crofford

**Affiliations:** Memphis, Tenn., Gynecologist to St. Joseph’s Hospital, Memphis Sanitarium, Etc.


For Daniel's Texas Medical Journal.

### SOME UNUSUAL CASES IN RECENT ABDOMINAL WORK.

BY T. J. CROFFORD, M. D., MEMPHIS, TENN.,
Gynecologist to St. Joseph's Hospital, Memphis Sanitarium, Etc.

CASE 1 was in the person of a virgin, aged 35, who had been a great sufferer for fifteen years. She was examined one year ago by myself and pronounced incurable, except by Tait's operation; but she fought this one year longer, was forced, however, by her constantly increasing invalidism, to yield. In this day of justifiable revolt against so many and such reckless abdominal operations, some go too far in the revolt.

This case illustrates the fact that there arise diseases of the appendages, even in the virgin, which cannot be cured by other means than the removal of the diseased structures by section. The recovery was prompt.

CASE 2. Mrs. A., aged 27, married, two children. Like the first case, was examined one year ago and pronounced incurable except by Tait's operation, so enlarged and adhered were the tubes and ovaries. She declined the operation and resorted to the use of electricity. There was no permanent relief. On last January an attack of peritonitis came near ending her life and determined her to have the operation performed as soon as practicable, notwithstanding more formidable adhesions which had now taken place, rendered the operation more difficult and hazardous.

This case illustrates the delusion of electricity and the dangers of delay when an operation is inevitable. There is more danger from one of these attacks of peritonitis, to which these cases are prone, than from the operation when skillfully done under the modern methods. She got well.

CASE 3 was an ovarian tumor weighing fifteen pounds; of interest in being of six years' growth, and so closely simulating a fibroid as to make diagnosis impossible. It was quite tense and filled with a fluid looking like pus. The case is interesting also on account of the almost universal adhesions; those of the anterior abdominal wall were quite strong. The omentum was spread out like a fan upon the surface, and the small intestines were so strongly attached as to require an enterorrhaphy after their separation from the tumor; but the point of greatest interest in the case is the unorthodox method of conducting the operation. Instead of tapping the cyst, as is the custom, the incision was extended up to the ensiform cartilage, preferring to separate the adhesions on all sides of the tumor, rather than its collapsed condition. Also, by this means, the danger of liberating the septic fluid in the peritoneal cavity was obviated. The advantages of these points in gaining time and safety are very great, much more than the dangers incurred by an extension of the incision to the ensiform cartilage.

CASE 4. Mrs. H., of Ark., aged 62 years, was the subject of a very large polycystic ovarian tumor (Figs. 1 and 2) of six years duration. It had been tapped seven times and she was proposing to have it again emptied when her husband came over to consult me upon the subject of its removal. I advised him to bring her at once to the sanitarium, without drawing off any of the fluid, preferring to operate as it was. The patient was much withered and exhausted from lugging around the enormous growth. The adhesions, as expected, were firm to the abdominal wall in front, so dense as to fall off the pivotal peritoneum and cause free hemorrhage from many places. The fluid was rapidly evacuated through the trocar and through openings torn into the sac from separated adhesions. The small pedicle was secured in the usual way. The tumor unquestionably received most of its nourishment through the adhesions, as the vessels entering through these were much larger than those of the pedicle. It will be remembered as the reason given for the occasional absence of a true pedicle in these tumors being a rotation or twisting, causing an atrophy and swerving of the pedicle, the nourishment coming

Fig. 2.

readily through the adhesions. The fluid filled three ordinary water buckets. The colloid material and sac added to this, making altogether seventy-five pounds in weight. The operation was done on the 28th of December last. She made an excellent recovery, and is since in all respects well.

The points of interest in this case were:

1. The size of the tumor.
2. The extent and density of the adhesions.
3. The smallness of the pedicle.
4. The length of time in existence and number of tappings.
5. The age and feebleness of the patient.

CASE 5. Miss K., aged 19, had been a sufferer from pelvic peritonitis for three years, having had many doctors and having swallowed many draughts of medicine, all to no relief. She called upon me. I began a study of her case. After excluding other causes, I concluded it to be due to disease of the vermiform appendix. Although no tumor could be defined at the McBurney point, the tender spot was here, and had been pretty much all the time. Upon the least exercise she would be compelled to assume the recumbent posture for a day or two, at least, on account of pain in the whole lower abdomen. I offered her abdominal section and removal of the appendix as a probable cure of her disease. The fact of her frequent attacks, great suffering, and fast yielding to the desire for morphine, induced me to urge and her to accept the operation, although we could not positively assure her of the correctness of the diagnosis. The incision was made over the McBurney point. The small intestines presented a very fiery and angry appearance, and for some twenty minutes or more, in vain looked for something to which the inflammatory condition present could be charged, not believing there could be such a thing as idiopathic peritonitis. The ovaries were examined for an offending cause, the tubes were investigated to see if there was a leak, and after weary of the search, back under the cæcum, a little point which looked slightly more swollen and fiery than the rest of the intestinal surface, was discovered, and whilst it did not present a formidable appearance, yet Dr. A. B. Holder, who has been assisting me in my abdominal work for the past two years, insisted that it would be best to remove the little point. I did so (Fig. 3) upon the ground that it was the most formidable point that could be found. There can be no

Fig. 3.

doubt but it was the appendix veriformis which the three years of inflammation had atrophied down to this extent. It weighed seven grains (Fig. 2). The operation was done on the 7th of last December. She never suffered a twinge of her old pains, and has gotten fat, well and strong.

I thought it might add to the interest of these cases to report them by contrast and to call attention to the fact that the old lady, 62 years old, in her emaciated and withered condition, lugging her 75 pound tumor, did not suffer and was not helpless at all comparable to the young lady, aged 19 years, with her tumor weighing only seven grains.

Points of interest in the case are:

1. The chronicity and length of time it had continued.

2. The helplessness, coupled with the youth of the patient, and the small size of the tumor.

## Figures and Tables

**Figure f1:**
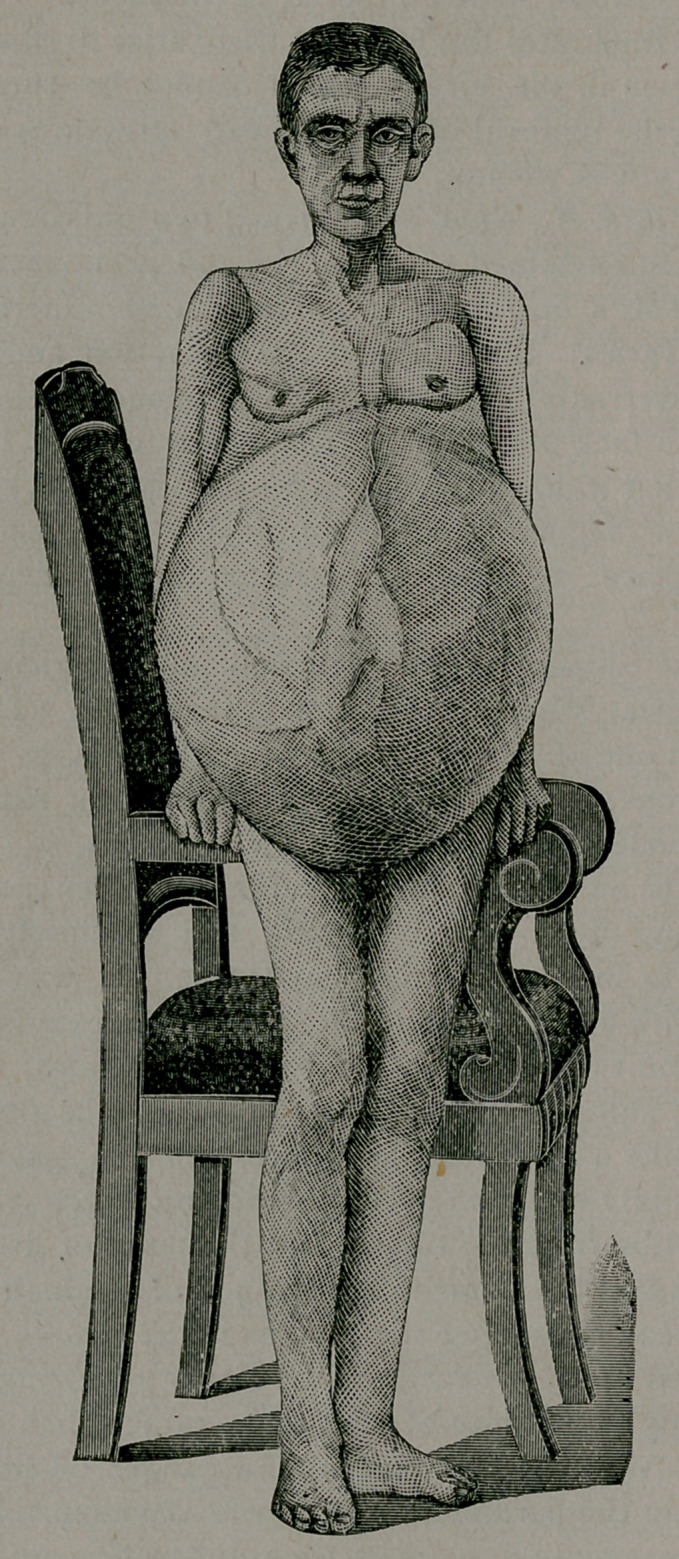


**Fig. 2. f2:**
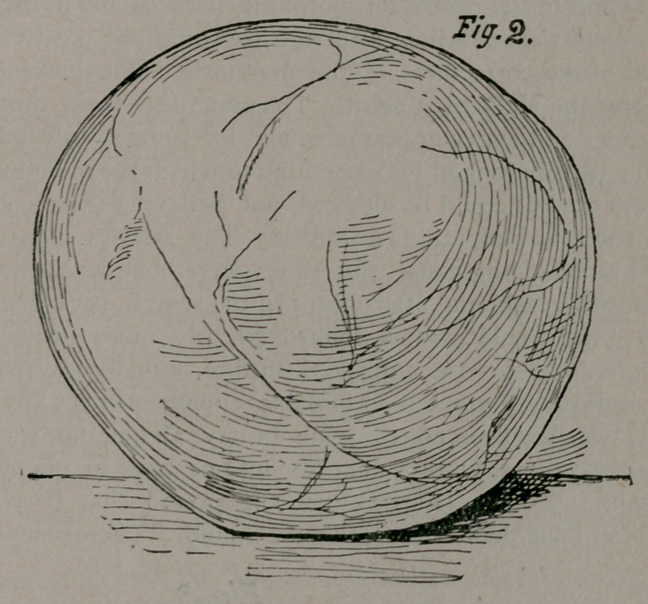


**Fig. 3. f3:**